# Study on the methodology of striae gravidarum severity evaluation

**DOI:** 10.1186/s12938-021-00945-w

**Published:** 2021-10-25

**Authors:** Hongyan Dai, Yangyang Liu, Yan Zhu, Yun Yu, Lin Meng

**Affiliations:** 1 Yanshan People’s Hospital of Wuxi City, Dacheng Road 1128, Wuxi, Jiangsu China; 2 College of Information and Communication Engineering, Nanjing Institute of Technology, Hongjing Avenue 1, Nanjing, Jiangsu China; 3 School of Bioengineering and Information, Nanjing Medical University, Longmian Avenue 101, Nanjing, Jiangsu China

**Keywords:** Striae gravidarum, Laser treatment, Evaluation parameters, Support vector machine

## Abstract

**Background:**

Striae gravidarum is a common occurrence in pregnancy and many women expect to prevent its development. At present, laser treatment has been used to improve the appearance of striae gravidarum, but the choice of laser type, treatment time, and frequency depend on the therapeutic effect. How to obtain an effective evaluation of striae gravidarum during and after treatment is very important. However, there is no unified evaluation parameter about striae gravidarum. In this paper, we studied the methodology evaluation of striae gravidarum severity. First, the laser therapeutic apparatus was selected as the experimental equipment and different striae gravidarum photos during treatment were obtained. Second, the subject evaluation parameters were chosen based on the literature research and the dermatologists’ guidance. Then, the striae gravidarum photos were divided into different groups by dermatologists based on these parameters. Finally, the objective detection parameters were designed based on the photos feature and subject evaluation parameters. Then, the objective detection parameters were used as the input of the support vector machine and the evaluation results were compared.

**Results:**

Based on the subject evaluation parameters, the experimental data could be divided into mild, moderate and severe groups. The experiment results showed that the striae gravidarum severity of two randomly patients were improved before and after treatment, which verified the validity of the parameters. In addition, the chosen objective detection parameters were different among different groups. With all the objective parameters as the support vector machine input, we could achieve the best recognition rate (82.71%) in the striae gravidarum severity classification. The four parameters (color difference, average density, average width, distribution area) calculated from the photos as the input could achieve acceptable accuracy (81.69%).

**Conclusions:**

The subject evaluation parameters and objective detection parameters proposed in this paper can be used to evaluate the striae gravidarum severity, which is of great significance for the construction of auxiliary diagnostic instrument for striae gravidarum treatment.

## Background

Striae gravidarum is a kind of striae distensae commonly observed in primigravid and obese women [1]. The changes in skin tension and hormone in the abdominal wall during the pregnancy lead to the rupture of elastic fibers of the abdominal skin [2]. The striae gravidarum presents as irregular purple or light red parallel and slightly concave expansion stripes in abdominal skin, and gradually fading into silver bright stripes [3]. Striae gravidarum brings both psychological burden and mental stress to pregnant women, leading a decrease of their life quality [4]. The occurrence and treatment have always been a worldwide concern in present medical research [5, 6]. Osman found that the occurrence of striae gravidarum was closely related to family history, excessive weight gain during pregnancy, and insufficient exercise [7]. The maternal age, basic body mass index, weight gain, and neonatal weight were also reported as risk factors of striae gravidarum [8]. Biological studies also suggested that stria gravidarum was related to the decrease of serum relaxin level [9]. However, there is no effective method to prevent the occurrence of striae gravidarum.

Once striae gravidarum appears, local drugs and instruments are commonly used to reduce its severity in clinics [10, 11]. A cream containing *Centella asiatica* had been found to be effective and it reduced most striae gravidarum, but such cream is not available in most countries [12]. Some researchers also found an anti-striae gravidarum cream could effectively prevent the formation of striae gravidarum, but it may cause side effects for long-term use [13]. Comparatively, the instrument is more convenient and safer, which have developed into a longtime treatment method for treating the postpartum striae gravidarum. For example, photon pigment regeneration instrument can emit ultraviolet waves with different energy-intensive ultraviolet photons to make the striae gravidarum color close to the normal skin [14]. However, after a period of time, some striae gravidarum may appear depigmentation and requiring maintenance treatment. Radiofrequency therapy instruments can make the dermis thicker by heating the location tissue and cooling the skin surface, which can reshape the collagen form under the skin and make the skin wrinkles disappear, but it needs more than one course of treatment to achieve a better curative effect [15]. Nowadays, laser therapy has been commonly used in skin diseases including expansion striae and striae gravidarum [16–18]. The CO_2_ laser is an infrared light with a wavelength of 10.6 μm, which penetrates deep tissue. Farahnaz compared CO_2_ laser therapeutic apparatus with glycolic acid and tretinoin compound cream in the treatment of 92 cases of striae gravidarum. He found that the CO_2_ laser therapeutic apparatus had a better effect in the striae gravidarum treatment [19].

Here, the CO_2_ laser therapeutic apparatus was selected as the experimental equipment, and then the striae gravidarum photos during the 12-week treatment course were recorded. Studies found that postpartum laser treatment is a good choice, but the choice of laser type, treatment start time, treatment frequency, and optimal course of treatment depend on the therapeutic effect, which needs to be further expanded [20]. How to obtain an effective evaluation of striae gravidarum during and after treatment is very important. However, there is no unified evaluation parameters about striae gravidarum. In some researches, subjective evaluation and relatively single objective parameters were used as observation parameters [13, 21, 22]. Keisha evaluated the striae gravidarum by scoring from 0 (no striae) to 4 (severe striae) [13]. We found that this evaluation method could only focus on the development or worsening of striae, and the color of stria gravidarum could be not observed. In Ref. [23], Davey divided the abdominal area into four parts. He scored each part from 0 to 2 and the total score was 0–8. This scoring system considered the different striae gravidarum areas, but only one parameter was scored. In the study on clinical effect of gold microneedle radio frequency, Chen evaluated the elasticity of skin at striae gravidarum at 2 months after the last treatment. The elasticity was an objective parameter, but it could not be observed directly [24].

Considering the multi-parameters and objective observation, we present the systematic evaluation of the striae severity according to the clinical characteristics of striae gravidarum in this paper. The CO_2_ laser therapeutic apparatus was selected as the experimental equipment. After we obtained different striae gravidarum photos before and during the treatment, the subject evaluation parameters were designed based on the clinical characteristics and the dermatologists’ guidance, including color, distribution, depth, elasticity, and roughness. According to these parameters, the striae gravidarum photos were divided into three groups (severe, moderate, and mild) by dermatologists. Moreover, two patients were randomly chosen and the evaluation of the striae gravidarum severity was done, in order to verify the validity of the subject evaluation parameters.

In addition, based on the photos feature and subjective evaluation parameters, the objective observation parameters were calculated. However, some subject evaluation parameters could not be calculated directly from the photo. We chose color difference, average density, average width, distribution area, and average elasticity as the objective observation parameters. In the experiment, different objective detection parameters were used as the input of the support vector machine and the evaluation results were compared [25, 26]. The results showed that all the four objective parameters as the support vector machine input could achieve the best recognition rate (82.71%) in the striae gravidarum severity classification. The four parameters (color difference, average density, average width, distribution area) calculated from the photos as the input could achieve acceptable accuracy (81.69%). The subject evaluation parameters and objective detection parameters proposed in this paper can be used to evaluate the striae gravidarum severity, which is of great significance for the construction of auxiliary diagnostic instrument for striae gravidarum treatment.

## Results

### Experimental data

**Fig. 1:**
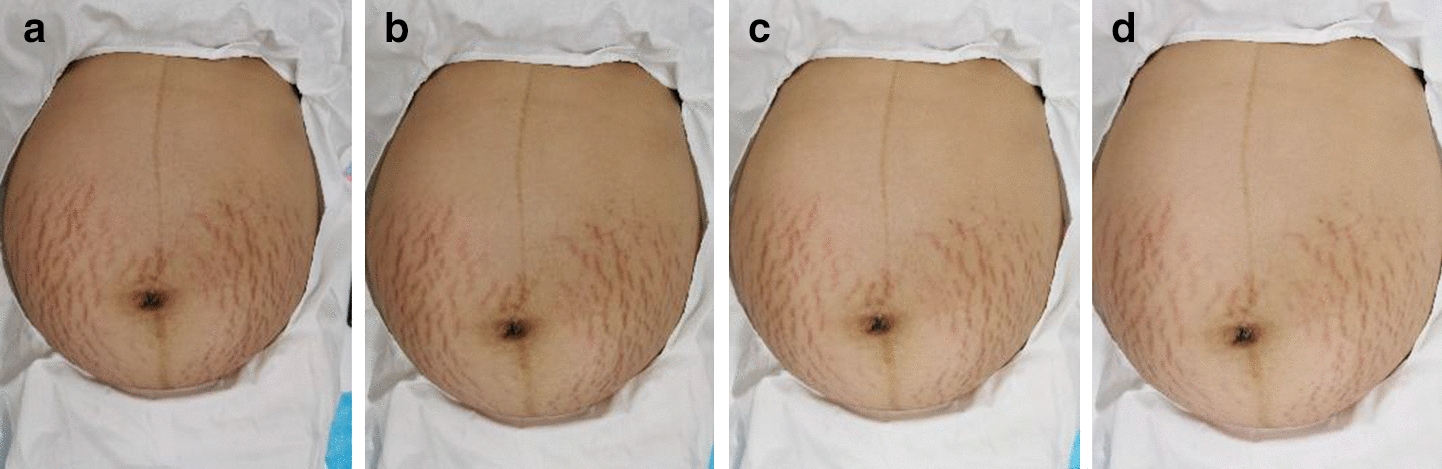
Striae gravidarum photos before treatment and during the treatment: **a** before treatment, **b** at the 2nd week, **c** at the 4th week, **d** at the 8th week

In this paper, 70 people participated in the treatment. Among them, 35 patients were photographed 5 times before and during the treatment. Other patients were photographed less than 5 times. Figure 1 gives an example of one woman’s striae gravidarum photos who was treated at 0, 2nd, 4th, 8th week. In Fig. 1, there was a slight improvement in the severity of striae gravidarum.

### Results based on subjective evaluation parameters

The subjective evaluation parameters were set including color, distribution, depth, elasticity, and roughness. The dermatologists scored each parameter (0, 1, 2, and 3 points), shown in Table 1. P1, P2, and P3 represented three randomly selected photos. D1, D2, and D3 represented the three dermatologists. The average of sum of 10–15 was severe, 5–9 was moderate and 1–4 was mild. Table 1 demonstrates how dermatologists classified the photos based on subject parameters.

**Table 1:** The subject evaluation of striae gravidarum severity

	Color	Distribution	Depth	Elasticity	Roughness	Sum	Average of sum	Category
P1								
D1	0	1	0	0	1	2	2.33	Mild (1–4)
D2	0	1	0	1	1	3		
D3	0	1	0	0	1	2		
P2								
D1	1	2	1	1	2	7	8	Moderate (5–9)
D2	2	2	1	1	2	8		
D3	2	2	1	2	2	9		
P3								
D1	2	3	3	2	2	13	13.33	Severe (10–15)
D2	3	3	2	3	3	14		
D3	3	3	2	2	3	13		

P1, P2, and P3: three randomly selected photos. D1, D2, and D3: three dermatologists

**Fig. 2:**
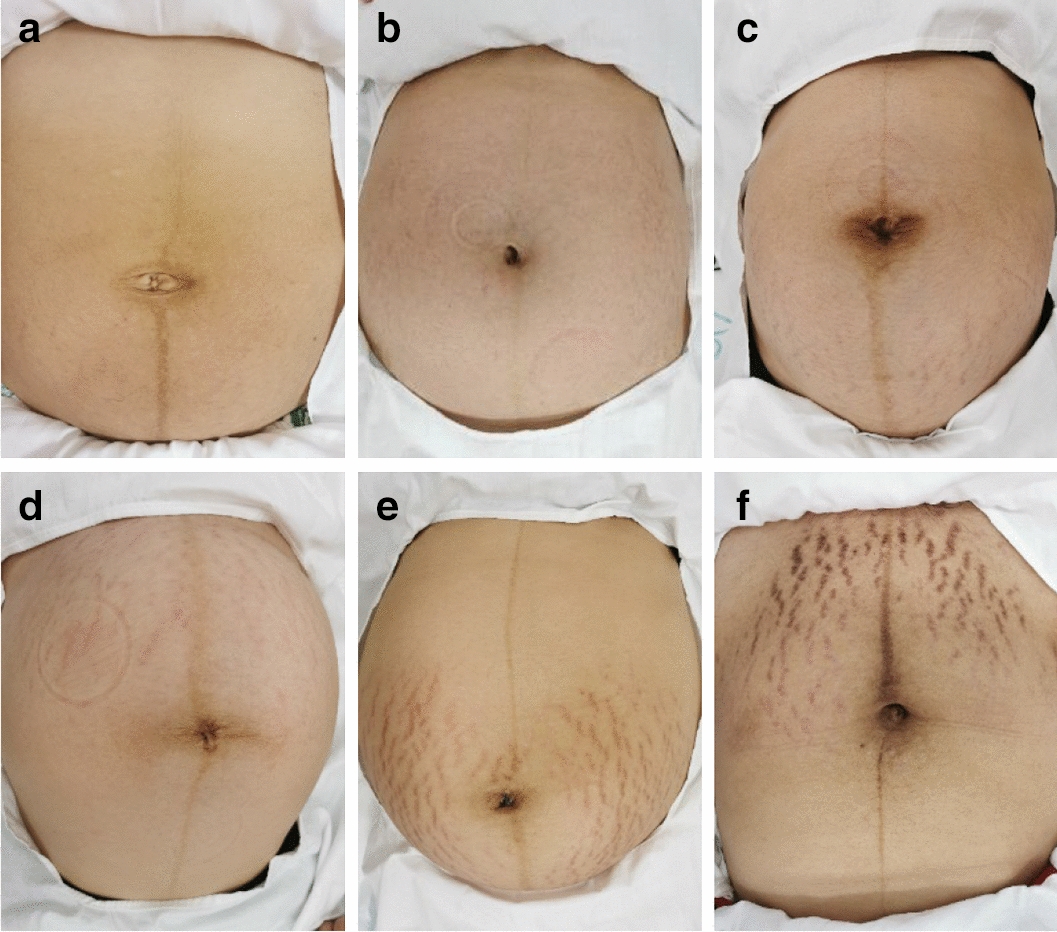
Striae gravidarum photos in different categories: **a**, **b** mild; **c**, **d** moderate; **e**, **f** severe

After subjective evaluation by dermatologists, all the photos were divided into three categories, including mild (80), moderate (76), and severe (86), as shown in Fig. 2 (each category for 2 cases). This research had obtained written informed consent of all subjects. The photos in different categories were different obviously.

**Fig. 3:**
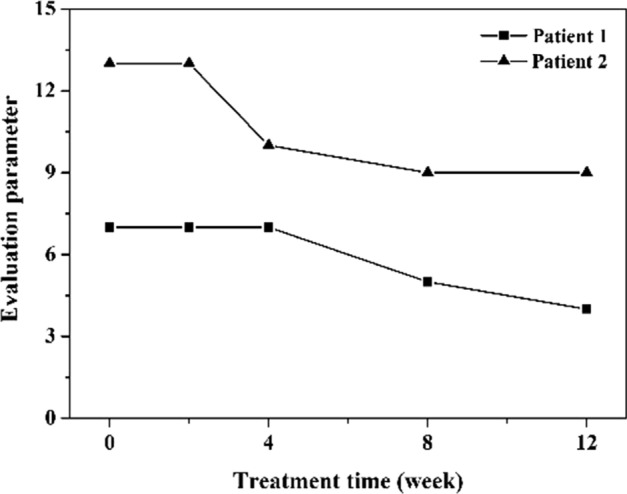
The subjective evaluation results during the treatment

To further prove the effectiveness of the subjective evaluation parameters, we chose the photos from two randomly selected patients at different stages of treatment. In Fig. 3, the striae gravidarum severity changes during the treatment were evaluated according to the subject evaluation parameters. The results showed that the striae gravidarum of the patient was improved after treatment, which were accepted by the patients. The therapeutic effects of different patients were different.

### Results based on the objective observation parameters

The objective observation parameters were set including color difference, average density, average width, distribution area, and average elasticity. In each group (mild, moderate, severe), all parameters were calculated in each photo. The average values of each parameter were obtained in each group, shown in Table 2. Comparing the average values of different categories, the objective detection parameters were different. However, we found that it was difficult to classify accurately based on a single objective observation parameter.

**Table 2:** Average value of striae gravidarum severity objective observation parameters

	Color difference	Average density	Average width	Distribution area	Average elasticity
Mild	0.021	0.135	2.89	0.15	0.86
Moderate	0.068	0.3584	3.8128	0.32	0.81
Severe	0.137	0.462	4.31	0.48	0.79

### Classification performance based on objective observation parameters

The 64 mild photos are regarded as training samples and 16 as testing samples. 61 moderate photos were regarded as training samples and 15 as testing samples. 69 severe photos were regarded as training samples and 17 as testing samples. The support vector machine classifier was used for mild, moderate, and severe classification. Different objective parameters were used as the input of the support vector machine and the classification performance is shown in Table 3. It showed that all the objective observation parameters as the classifier input could achieve the best recognition rate (82.71%). The elasticity parameter should be collected by the specific instrument. If we do not have the instrument, the four parameters (color difference, average density, average width, and distribution area) as the input could achieve acceptable accuracy (81.69%). In Ref. [23], it was found color was a very important parameter. Therefore, we compared the classification results of color difference and other parameters as the input. The experimental results show that the recognition rates of three parameters as the input were smaller.

**Table 3:** Recognition rate based on objective observation parameters

Classifier input	Recognition rate/%
Color difference + average width + distribution area	75.85
Color difference + average density + distribution area	77.31
Color difference + average density + average width	76.11
Color difference + average density + average width + distribution area	81.69
Color difference + average density + average width + distribution area + average elasticity	82.71

## Discussion

Striae gravidarum are atrophic linear scars commonly seen in the connective tissues of pregnant women. They have been reported to occur in most pregnant women and usually occur in the abdomen, but can also develop in the hips, breasts, thigh, groins, and armpits [27]. Nowadays, many attempts have been made to identify risk factors, prevention methods, and treatments. For prevention of striae gravidarum, the current most effective therapies include some cream combined instruments such as laser equipment [28]. Laser treatment appears to yield on average greater way of improvement and in a much shorter time than topical treatments.

Studies have shown that red light can stimulate cells, especially fibroblasts, to repair tissue. It is widely used in the treatment of skin diseases, common skin ulcers, sores, and herpes zoster [29]. When the red light is applied to the repair of striae gravidarum, the epidermis will selectively absorb red light and stimulate the damaged collagen cortex to constantly repair itself, to improve the skin condition. Yi studied the clinical effect of red light in the treatment of striae gravidarum [30]. After three cycles of treatment, the total effective rate was 87.50%. The application of red light on striae gravidarum repair is still under exploration, and more experiments are needed to verify the efficacy. In this paper, CO_2_ laser therapeutic apparatus was selected to obtain the striae gravidarum photos before treatment and after 2, 4, 8, and 12 weeks of treatment.

It is very important to evaluate striae gravidarum effectively during and after treatment. In some striae gravidarum research, the scoring method was often used to evaluate the overall situation of striae gravidarum. However, these methods were usually based on a single severity score, which did not involve specific parameters such as the color, depth, and elasticity of striae gravidarum, and there was no clear objective observation index. In this paper, subjective evaluation and objective observation parameters were designed, respectively. The subjective evaluation parameters were chosen as color, distribution, depth, elasticity, and roughness. According to these parameters, the striae gravidarum photos were divided into three groups by dermatologists. Based on the photo features and subjective evaluation parameters, the objective parameters were designed as color difference, average density, average width, distribution area, and average elasticity. Roughness and depth could not be calculated from the photo, then average density and average width were used as objective parameters. Based on the subjective evaluation results, a support vector machine evaluation classifier was constructed to objectively evaluate the striae gravidarum severity.

**Fig. 4:**
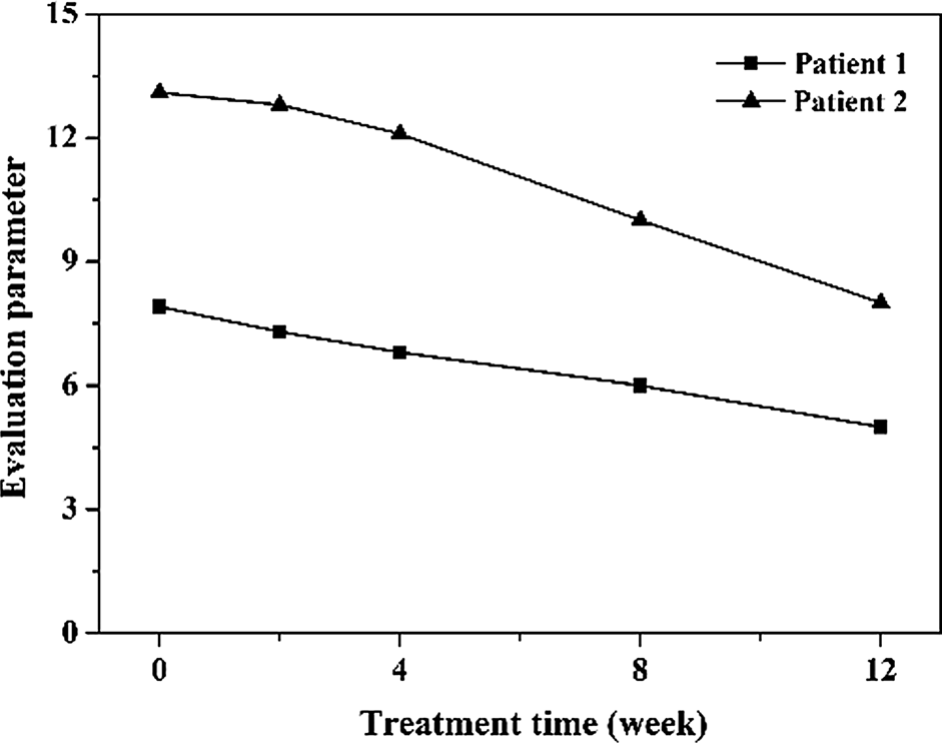
The objective evaluation results during the treatment

To further prove the effectiveness of the objective parameters, we also chose the photos form the two patients in Fig. 3. The striae gravidarum severity changes during the treatment were evaluated according to the objective observation parameters in Fig. 4. To facilitate comparison, each objective observation parameter was normalized to 0–3, and the average of sum was calculated. The results showed that the results of objective evaluation were consistent with that of subjective evaluation. The curves indicated that the striae gravidarum of the patient was significantly improved after treatment. Compared with the subjective evaluation, the curves of objective evaluation changed gently. However, for convenient calculation, each objective parameter was only normalized to 0–3 and summed. In the next study, we will conduct accurate mathematical models for objective evaluation, rather than simple summation or support vector machine.

## Conclusions

Striae gravidarum is a common form of gestational change that can be a substantial source of distress. Various therapies have been studied to reduce the severity of striae gravidarum. How to find a more effective treatment is a difficult problem and further results from large, randomized-controlled studies are required. Therefore, effective evaluation of striae gravidarum is very important during the treatment. In this paper, we studied on the methodology of striae gravidarum severity evaluation. We draw up the subjective evaluation parameters on the clinical characteristics and the dermatologists’ guidance. Based on the subject evaluation parameters, the experimental data could be divided into mild, moderate and severe groups, and it was found the striae gravidarum severity of two randomly patients were improved before and after treatment. Next, the objective observation parameters were designed, including Average color, Average density, Average width, Average distribution, and Average elasticity. As the feature input of the support vector machine, all the objective observation parameters from striae gravidarum photos could achieve the best recognition rate in the severity classification (82.71%). The four parameters (color difference, average density, average width, and distribution area) as the input could achieve acceptable accuracy (82.71%). Although there were some differences between subjective parameters and objective observation parameters, the evaluation results from the patients during the treatment of objective evaluation were consistent with that of subjective evaluation. The subject evaluation parameters and objective detection parameters proposed in this paper were effective and used to evaluate the striae gravidarum severity, which is of great significance for the construction of auxiliary diagnostic instrument for striae gravidarum treatment.

## Methods

### Experimental data

Women were recruited from the Yanshan People’s Hospital of Wuxi City. The average age was 29.75 years (25–39 years). The production days ranged from 47 to 233 days with an average of 110.5 days (about 12–15 weeks of pregnancy). They have had no cancer, infectious disease, blood disease, lipoma, hemangioma, vascular embolism, pregnancy, and other treatment in the past 6 months. All patients have informed the parturient of the matters needing attention in the treatment process and obtain the informed consent of the parturient. The study was approved by the Yanshan People’s Hospital of Wuxi City Ethics Committee. Before and after the treatment, the original photos of the abdominal striae gravidarum were taken. After subjective evaluation of each photo, data were obtained including 80 mild cases, 76 moderate cases, and 86 severe cases.

### Treatment system and methods

CO_2_ laser therapeutic instruments (JZ-1-1A, Chengdu Guoxiong Optoelectronic Technology Co., Ltd) were used in this paper. It has a wavelength of 10.6 μm ± 0.1 μm and 450VA rated input power. All patients received radiation therapy under the guidance of professionals. Before treatment, the patients were informed of the implementation steps and precautions of laser irradiation treatment. The instrument light source emitted red light and directly irradiated the treated part without shielding. Each treatment lasted for 30 min (twice a day) and every 4 weeks was a cycle. After 3 cycles, the treatment efficacy of the treatment was evaluated. Most patients were photographed before treatment and at the 2nd, 4th, 8th, and 12th weeks of treatment. The subjective and objective evaluation of striae gravidarum severity were done in each photo.

### Subjective evaluation parameters calculation

According to the clinical characteristics and the dermatologists’ guidance, the subjective evaluation parameters were set including color, distribution, depth, elasticity, and roughness. Three dermatologists (each with 3–5 years’ experience) who did not participate in the treatment scored the photos of each patient before and during the treatment. They scored each parameter (0–3 points). 0 represented normal skin condition (no striae gravidarum) and 3 represented severe skin condition (obvious striae gravidarum). In each photo, the sum of all parameters was calculated and the average of sum was obtained. The average of sum of 10–15 was severe, 5–9 was moderate and 1–4 was mild. If it was less than or equal to 1, the evaluation was invalid.

### Objective observation parameters calculation

Based on subjective evaluation parameters, the objective observation parameters were set to judge the treatment effect, including color difference, average density, average width, distribution area, and average elasticity. Although we hope the parameters of objective evaluation were consistent with that of subjective evaluation, some subject evaluation parameters could not be calculated directly from the photo. Roughness and depth could not be calculated from the photo, then average density and average width were used as objective parameters. Color difference represents the difference between the difference of gray average value between the skin region and striae gravidarum region. Distribution area represents the distribution area index of striae gravidarum in the abdomen. The elasticity parameter was collected by the specific instrument.

**Fig. 5:**
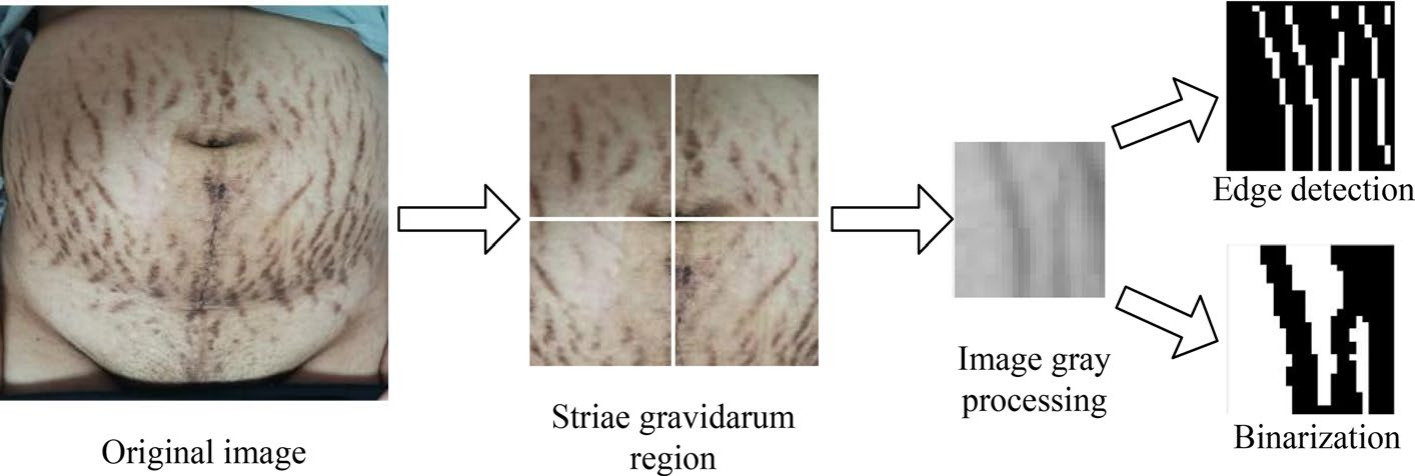
Striae gravidarum photo processing

Objective observation parameters were calculated after stria gravidarum photo pretreatment. Specifically, skin region (SR) was segmented based on skin color detection algorithm [31]. The largest square was segmented with the navel as the center, named striae gravidarum region (SGR). Different evaluation parameters were calculated after image processing in SGR, including binarization and edge detection, shown in Fig. 5:

1. In the SGR, graying and binarization operations (adaptive threshold) were done and the gray average value of the pixels in the black region and the white region were calculated. The region with a gray value similar to skin color was defined as *R*_S_, the other region was *R*_SG_. The average gray values of the corresponding regions were *G*_s_ and *G*_SG_, respectively. Then, the color difference (*C*) was calculated by *C* = abs(*G*_SG_ − *G*_S_)/255.
2. The number of pixels in the *R*_S_ area was *N*_S_, and the number of pixels in the *R*_SG_ area was *N*_SG_. Average density (*D*) was calculated by *D* = *N*_SG_/(*N*_SG_ + *N*_S_).
3. Edge detection (Canny edge detection) and image smoothing were performed in the SGR, then the number of edges was calculated, named *N*_e_. Average width (*W*) was obtained by *W* = 2 * *N*_SG_/*N*_e_.
4. The SGR was divided into four areas with the navel as the center. The average density *D*_1_, *D*_2_, *D*_3_, *D*_4_ of each area was calculated, which was compared with 0.4 (empirical value). The number greater than 0.4 was named *N*. Distribution area (*A*) was achieved by *A* = *N* * 0.25.
5. Average elasticity was measured by skin elasticity tester (CU-TOMETER SEM 575, CK company, Germany) [32]. 10 parameters from *R*0 to *R*9 were obtained from skin elasticity tester, in which *R*2 represented total elasticity index and *R*5 represented skin plasticity recovery index. The closer the index was to 1, the better the skin elasticity was. *R*2 was used as the average elasticity (*E*) parameter.

### Striae gravidarum severity evaluation based on support vector machine

Support vector machine was used to evaluate striae gravidarum and classify mild, moderate, and severe [33]. Support vector machine uses maximal margin to separate the two known labeled data according to a hyperplane and support vectors are defined as those data points that are closest to the hyperplane. The hyperplane that maximizes the margin is as follows:

1 $$ w \cdot x + b = 0, $$

where *x* denotes the features set of input samples. We define the distance from the support vectors to the hyperplane as *d*, and the distance from other points to the hyperplane is greater than *d*:

2 $$ d = \frac{{\left| {w^{{\text{T}}} } \right| + b}}{\left\| w \right\|}. $$

Equation (2) can be transformed into the optimization problem as follows:

3 $$ \Phi (w) = \frac{1}{2}\left\| w \right\|^{2} = \frac{1}{2}(w \cdot w),{\text{s}}.{\text{t}}.\quad y_{i} [(w \cdot x_{i} ) + b] - 1 \ge 0, $$

where (*x*_*i*,_ *y*_*i*_) ($i = 1,2,...,N$) denotes a training labeled sample set. In order to solve the optimization problem, the following Lagrange function is defined:

4 $$ L(w,b,\alpha ) = \frac{1}{2}(w \cdot w) - \sum\limits_{i = 1}^{n} {\alpha_{i} } \left\{ {y_{i} [(w \cdot x_{i} ) + b] - 1} \right\}, $$

where $\alpha_{i} \ge 0$ denotes the positive Lagrange multiplier, *L* can be transformed as follows:

5 $$ \max Q(\alpha ) = \sum\limits_{i = 1}^{n} {\alpha_{i} } - \frac{1}{2}\sum\limits_{j = 1}^{n} {\alpha_{i} \alpha_{j} y_{i} y_{j} (x_{i} \cdot x_{j} )} ,{\text{s}}.{\text{t}}.\quad \sum\limits_{i = 1}^{n} {\alpha_{i} y_{i} = 0,\;\alpha_{i} \ge 0.} $$

Let $\alpha_{i}^{*}$ denote the optimization value, the *w* and *b* can be calculated as follows:

6 $$ w^{*} = \sum\limits_{i = 1}^{n} {\alpha_{i}^{*} } y_{i} x_{i} , $$

7 $$ y_{i} [(w \cdot x_{i} ) + b] - 1) = 0. $$

The linear kernel function was used in support vector machine in this paper.

## Data Availability

Data sharing does not apply to this article.

## Abbreviations

SR: Skin region
SGR: Striae gravidarum region
