# Correction to: Study on the methodology of striae gravidarum severity evaluation

**DOI:** 10.1186/s12938-021-00964-7

**Published:** 2021-12-17

**Authors:** Hongyan Dai, Yangyang Liu, Yan Zhu, Yun Yu, Lin Meng

**Affiliations:** 1 Xishan People’s Hospital of Wuxi City, Dacheng Road 1128, Wuxi, Jiangsu China; 2 College of Information and Communication Engineering, Nanjing Institute of Technology, Hongjing Avenue 1, Nanjing, Jiangsu China; 3 School of Bioengineering and Information, Nanjing Medical University, Longmian Avenue 101, Nanjing, Jiangsu China

## Correction to: BioMed Eng OnLine (2021) 20:10 10.1186/s12938-021-00945-w

Following publication of the original article [1], the author noticed an error in the first affiliation. The unit name be “Xishan People’s Hospital of Wuxi City, Dacheng Road 1128, Wuxi, Jiangsu, China” instead of “Yanshan People’s Hospital of Wuxi City, Dacheng Road 1128, Wuxi, Jiangsu, China.” This corrected affiliation appears in the following texts:

In Methods section, the first sentence should read, “Women were recruited from the Xishan People’s Hospital of Wuxi City” instead of “Women were recruited from the Yanshan People’s Hospital of Wuxi City”.

In the same section, the fifth sentence should read, “The study was approved by the Xishan People’s Hospital of Wuxi City Ethics Committee” instead of “The study was approved by the Yanshan People’s Hospital of Wuxi City Ethics Committee”.

Under Declarations, the first sentence in “Ethics approval and consent to participate” should be “This study was approved by the Xishan People’s Hospital of Wuxi City Ethics Committee” instead of “This study was approved by the Yanshan People’s Hospital of Wuxi City Ethics Committee”.

The original article has been corrected.
